# The microvesicle/CD36 complex triggers a prothrombotic phenotype in patients with non‐valvular atrial fibrillation

**DOI:** 10.1111/jcmm.15311

**Published:** 2020-06-08

**Authors:** Hua Wang, Nian‐Peng Song, Jian‐Ping Li, Zhi‐Hao Wang, Yun Ti, Yi‐Hui Li, Wei Zhang, Ming Zhong

**Affiliations:** ^1^ Department of Cardiology Yantai Yuhuangding Hospital Qingdao Medical College Qingdao University Yantai China; ^2^ The Key Laboratory of Cardiovascular Remodeling and Function Research Chinese Ministry of Education Chinese National Health Commission and Chinese Academy of Medical Sciences The State and Shandong Province Joint Key Laboratory of Translational Cardiovascular Medicine Department of Cardiology Qilu Hospital of Shandong University Jinan China; ^3^ Department of Geriatric Medicine Key Laboratory of Cardiovascular Proteomics of Shandong Province Qilu Hospital of Shandong University Jinan China; ^4^ Department of Critical Care Medicine Qilu Hospital of Shandong University Jinan China

**Keywords:** CD36, non‐valvular atrial fibrillation, platelet activation, platelet microvesicles

## Abstract

The mechanisms responsible for platelet activation, the prothrombotic state, in non‐valvular atrial fibrillation (NVAF) are still obscure. Microvesicles (MVs) can transfer various messages to target cells and may be helpful for exploring the detailed mechanisms. We aimed to investigate the possible mechanisms by which proatherogenic factors of NVAF contribute to platelet activation. Two hundred and ten patients with NVAF were stratified as being at ‘low to moderate risk’ or ‘high risk’ for stroke according to the CHADS2 score. Levels of platelet‐derived MVs (PMVs) and platelet activation were examined. CD36‐positive or CD36‐deficient human platelets were stimulated by MVs isolated from NVAF patients with or without various inhibitors in vitro*.* Levels of PMVs and platelet activation markers enhanced significantly in high‐risk patients. The MVs isolated from plasma of NVAF patients bound to platelet CD36 and activated platelets by phosphorylating the mitogen‐activated protein kinase 4/Jun N‐terminal kinase 2 (MKK4/JNK2) pathways. However, CD36 deficiency protected against MV‐induced activation of platelets. We reveal a possible mechanism of platelet activation in NVAF and suggest that the platelet CD36 might be an effective target in preventing the prothrombotic state in NVAF.

## 1 INTRODUCTION

Non‐valvular atrial fibrillation (NVAF) is a major cause of stroke. Although warfarin is the mainstay of antithrombotic therapy for NVAF patients in Asian countries, 3.06% of patients per year on warfarin still experience stroke or systemic embolism in Asians.^1^ The mechanisms underlying thromboembolism in NVAF patients are still obscure. Platelets provide a catalytic surface for coagulation and bind coagulation factors. In addition, approximately 56% of NVAF patients have concomitant ischaemic heart disease.^2^ Concomitant use of antiplatelet therapy and oral anticoagulation was recommended in current guidelines in NVAF patients with ischaemic heart disease.^3^

However, antiplatelet therapy in NVAF patients has been challenged. Compared with anticoagulant monotherapy, the combination therapy of antiplatelet therapy and oral anticoagulation increases risk of bleeding without reduction in risk of thromboembolism.^4^ Insufficiency of the present antiplatelet therapy may be associated with the diversity of platelet activation pathways. The molecular mechanisms that underlie platelet activation in NVAF are poorly defined but may involve the aetiology of NVAF.

The potential effects of inflammation, oxidative stress and metabolic disorders in the NVAF‐related prothrombotic state have aroused much attention.^5^ However, these risk factors have not been integrated together in targeting prevention of NVAF‐related platelet activation. To determine a vector exhibiting all these proatherogenic factors in NVAF, we can study the potential mechanisms of platelet activation and explore new, effective targets for preventing thrombosis in NVAF. Recently, studies of microvesicles (MVs) have enlightened us.^6^, ^7^, ^8^

Circulating microvesicles (MVs), submicrometer vesicles formed by activated cells, can act as messengers delivering a variety of cargos, including cell surface receptors, proinflammatory cytokines and signature proteomes, to target cells.^9^, ^10^ Circulating levels of procoagulant MVs, result from integrated actions of oxidative stress and inflammation, are increased in patients with NVAF.^6^, ^11^, ^12^ In a recent study, investigators found that MVs isolated from plasma of polyangiitis patients can activate platelets.^13^ Thus, we speculated that MVs may be important mediators between pathogenic factors and platelet activation in NVAF. Phosphatidylserine (PS) on the surface of MVs could be a ligand of CD36, thus generating a MV‐CD36 complex.^14^ Chen et al^15^ showed that platelet CD36 mediates mitogen‐activated protein kinase (MAPK) activation induced by oxidized low‐density lipoprotein (oxLDL). We previously showed that platelet‐derived microvesicles (PMVs) generated in response to oxidative insult could activate CD36/mitogen‐activated protein kinase 4/Jun N‐terminal kinase 2 (CD36/MKK4/JNK2) signal pathway and lead to platelet activation.^16^ Whether the same pathway was also activated in the context of NVAF is unclear.

In the study, we sought to determine whether the MVs, vectors of integrating all the proatherogenic factors in NVAF, could be endogenous CD36 ligands that transmit an activating signal to platelets to induce prothrombotic phenotype and whether the PMV‐CD36 complex is an effective target for treating NVAF‐related thrombosis.

## 2 METHODS

### 2.1 Study design

A case‐control study was carried out. The study conformed to the Declaration of Helsinki and was approved by the institutional ethics committee. Written informed consent was obtained from each subject. We included 210 patients with persistent or paroxysmal NVAF (mean age 62.23 ± 11.47 years; 127 men) and 35 healthy controls (mean age 55.71 ± 7.43; 17 men). Patients were classified as being at ‘low to moderate risk’ or ‘high risk’ for stroke according to the CHADS2 risk scheme.^3^ Patients with CHADS2 score 0 or 1 were considered at ‘low to moderate risk’ and those with score ≥ 2 at ‘high risk’. To minimize the effect of medication, all the subjects had not taken aspirin or non‐steroidal anti‐inflammatory drugs (NSAIDs) for 1 week before blood samples were collected. Basic characteristics and blood examination of all patients and healthy controls were recorded. The expression of platelet integrin α_IIb_β_3_ and CD36 was detected by flow cytometry. Measurement of oxidized low‐density lipoprotein (oxLDL), interleukin‐6 (IL‐6), 8‐iso‐prostaglandin F2α (8‐iso‐PGF2α) and soluble P‐selectin was tested by enzyme‐linked immunosorbent assay (ELISA). Plasma levels of PMVs or PS‐positive PMVs were detected by ELISA and flow cytometry. For MV stimulation experiments, CD36‐positive or CD36‐deficient resting platelet suspensions were exposed to MVs collected from AF patients. Platelet integrin α_IIb_β_3_, CD40L and CD36 were evaluated by flow cytometry. P‐selectin was tested by ELISA. Inhibitory strategies with Annexin V and specific antibodies were applied to investigate the role of PS, CD36 or MKK4/JNK2 signalling pathway in the interactions. Phosphorylation of MKK4 and JNK was analysed by Western blotting.

### 2.2 Reagents

Biotin‐conjugated anti‐CD41 monoclonal antibody was from Abcam. Recombinant human Annexin V was from Enzo Life Sciences. SP600125 was from Calbiochem. The antibodies rabbit anti‐MKK4, p‐MKK4, c‐JNK and mouse anti‐p‐JNK were from Cell Signaling Technology. Phycoerythrin (PE)‐conjugated anti‐CD36 (clone CB38), PE‐conjugated CD36 isotype control (clone G155‐228), PE‐cy™5‐conjugated mouse anti‐CD41a antibody (clone HIP8) and fluorescein isothiocyanate (FITC)‐conjugated PAC‐1 were from BD Biosciences/Pharmingen. The 8‐iso‐prostaglandin‐F2α (8‐iso‐PG‐F2α) enzyme immunoassay (EIA) kit was from Cayman Chemical. The p‐selectin ELISA kit was from R&D Systems. The MV‐capture antibody AD‐1 was originally raised against human membrane‐bound liver alkaline phosphatase isolated from patients with liver cancer.^10^, ^11^ The hybridoma cells were cultured in RPMI 1640 medium containing 10% FBS to produce antibody.

### 2.3 Laboratory analysis

The expression of platelet integrin α_IIb_β_3_ (PAC‐1) and CD36 of subjects was detected by flow cytometry. Citrated plasma was obtained by centrifugation at 3000 *g* for 10 minutes. Immediately after double centrifugation of whole blood for 10 minutes at 3000 *g*, the plasma was centrifuged for 3 minutes at 12 000 *g* to prepare platelet‐free plasma (PFP). Measurement of oxLDL, IL‐6, 8‐iso‐PGF2α and soluble P‐selectin involved ELISA with commercial reagents (Uscn life science).

### 2.4 ELISA quantification of PMVs

Quantification of PMVs in PFP involved use of the home‐made ELISA as described.^16^, ^17^, ^18^, ^19^ Briefly, the MVs were captured on the surface of the ELISA plate coated with AD‐1. Then, the PMV detection was achieved by estimating the amount of CD41‐positive MVs. The plate was incubated with biotin‐conjugated mouse anti‐human CD41 monoclonal antibody (Abcam) at 37°C for 2 hour and then with horseradish peroxidase‐conjugated streptavidin (R&D Systems) for 30 minutes. The linear absorbance was read at 450 nm by a microplate reader (Thermo Scientific) after the addition of substrate.

### 2.5 ELISA quantification of annexin V‐positive PMVs

Quantification of Annexin V‐positive PMVs involved use of a home‐made ELISA as described with minor modification.^16^, ^20^ The PFP was recalcified by addition of 50 mmol/L CaCl_2_ immediately before laying into the wells of the plate previously coated with Annexin V (for capture of PS present on MVs). Then, the PMV detection was achieved by estimating the amount of CD41‐positive MVs as described above.

### 2.6 Platelet isolation

Platelets were prepared as previously described.^16^ The healthy volunteers had not taken any medication for 14 days. Venous blood was collected in 0.109 mol/L sodium citrate under minimal tourniquet pressure using a sterile 22‐gauge needle. Platelet‐rich plasma (PRP) was obtained by centrifugation at 120 *g* for 10 minutes. Washed platelets were isolated from PRP after centrifugation at 800 *g* for 10 minutes in the presence of 100 nmol/L prostaglandin E_1_ (PG‐E_1,_ Sigma‐Aldrich) and were resuspended in modified Tyrode's buffer [137 mmol/L NaCl, 2.7 mmol/L KCl, 12 mmol/L NaHCO_3_, 0.4 mmol/L NaH_2_PO_4_, 5 mmol/L HEPES, 0.1% glucose and 0.35% bovine serum albumin (BSA), pH7.2] with 100 nmol/L PG‐E_1._ Platelet concentration was adjusted to 1 × 10^6^/mL and then used at once.

### 2.7 Isolation of MVs from platelet supernatant and plasma

For isolation of MVs from the plasma, PFP of untreated NVAF patients was centrifuged at 15 000 *g* for 1 hour in 4°C to precipitate MVs. Then, the washed MVs from NVAF patients were characterized by flow cytometry by staining with PEcy5‐conjugated anti‐CD41a antibody and FITC‐conjugated Annexin V in binding buffer (10 mmol/L Hepes/NaOH, 140 mmol/L NaCl, 2.5 mmol/L CaCl_2_, pH 7.4) for 15 minutes at room temperature in the dark.

### 2.8 Platelet functional studies

For MV stimulation experiments, CD36‐positive or CD36‐deficient platelet suspension (1 × 10^6^/mL) was stimulated with 30 µg/mL MVs from NVAF patients for various times as indicated and used for subsequent experiments.

#### 2.8.1 Flow cytometry of platelet activation and CD36 expression

Expression of platelet integrin α_IIb_β_3_, CD40L and CD36 was detected by flow cytometry. The platelet suspension (2.5 µL) treated with MVs was incubated with 5 µL PEcy5‐conjugated anti‐CD41a antibody and 5 µL FITC‐conjugated PAC‐1 antibody (for activated platelet integrin α_IIb_β_3_) or PE‐conjugated anti‐CD40L antibody in the dark for 15 minutes. For CD36 quantification, the platelet suspension was incubated with 5 µL PEcy5‐conjugated anti‐CD41a antibody and 5 µL PE‐conjugated anti‐CD36 antibody or isotype‐matched control IgG. In some studies, before stimulation with MVs, platelets were pre‐incubated with an anti‐CD36 antibody, JNK inhibitor SP600125 or pre‐incubated with Annexin V to mask the PS on surface of MVs in binding buffer (10 mmol/L Hepes/NaOH, 140 mmol/L NaCl, 2.5 mmol/L CaCl_2_, pH 7.4).

#### 2.8.2 Immunoassay for soluble P‐selectin

After the above treatment, the platelet‐MV mixtures were centrifuged at 3000 *g* for 20 minutes twice. The last supernatant was used for detection of soluble P‐selectin.

#### 2.8.3 Western blot analysis of phosphorylation of MKK4 and JNK

Cell lysates were subjected to immunoblotting as previously described ^16^ with antibodies against phospho‐MKK4 (p‐MKK4) or total MKK4 and phospho‐JNK (p‐JNK) or total JNK (Cell Signaling Technology, Danvers, MA), followed by anti‐IgG horseradish peroxidase‐conjugated secondary antibody.

### 2.9 Statistical analysis

Data are expressed as mean ± SD (SE). Comparison among groups involved chi‐squared test (for categorical data) or ANOVA with post hoc least significant differences or Dunnett's *t* test (for continuous data). *P* < .05 was considered statistically significant. Analyses involved use of SPSS 16.0 (SPSS Inc).

## 3 RESULTS

### 3.1 Patient characteristics

#### 3.1.1 Characteristics of NVAF patients and healthy controls: relationship to stroke risk stratified according to the CHADS2 score

Of the 210 NVAF patients, 113 (53.81%) were at high risk of stroke. By definition of CHADS2 score, the high‐risk NVAF patients were older and more likely to have congestive heart failure, hypertension, diabetes mellitus or history of stroke than those at low to moderate risk or healthy controls (*P* < .001). Furthermore, systolic blood pressure, fasting blood glucose, creatinine and white blood cell counts were higher in patients at high risk compared with controls (*P* < .01) (Table 1).

**Table 1:** Characteristics of non‐valvular AF patients and healthy controls: relationship to stroke risk stratified by CHADS2 score

	Healthy controls (n = 35)	NVAF patients with low to moderate risk (n = 97)	NVAF patients with high risk (n = 113)
Age in years	55.71 ± 7.43	57.05 ± 10.41	66.68 ± 10.46***, †††
Male gender, n (%)	17(48.57%)	65(67.01%)	62(54.87%)
BMI	24.91 ± 4.97	25.81 ± 4.54	26.55 ± 3.72
WHR	0.87 ± 0.05	0.90 ± 0.05**	0.92 ± 0.06***
SBP	120.52 ± 11.38	131.09 ± 17.55**	141.83 ± 19.63***, †††
DBP	79.82 ± 8.66	79.04 ± 12.79	83.46 ± 14.23
Glucose (mmol/L)	5.20 ± 0.40	5.33 ± 1.52	6.10 ± 1.78***, ††
Cholesterol (mmol/L)	4.68 ± 0.68	4.78 ± 1.18	4.56 ± 1.15
TG (mmol/L)	1.34 ± 0.78	1.61 ± 1.02	1.50 ± 0.89
LDL‐C (mmol/L)	2.44 ± 0.47	2.72 ± 0.91	2.67 ± 0.83
HDL‐C (mmol/L)	1.34 ± 0.29	1.21 ± 0.29	1.19 ± 0.44*
Cr (mmol/L)	69.26 ± 12.76	75.84 ± 16.74	92.38 ± 53.87**, ††
WBC (×10^9^/L)	5.30 ± 0.80	6.17 ± 1.65	6.91 ± 1.91**, ††
Platelets (×10^9^/L)	206.27 ± 35.68	202.65 ± 82.44	191.5 ± 64.25
LVEF	0.62 ± 0.06	0.61 ± 0.07	0.56 ± 0.11**, †
E/E’	5.04 ± 1.02	7.04 ± 3.09***	8.86 ± 3.63***, †
Plasma markers			
oxLDL (µg/mL)	1.21 ± 0.88	2.03 ± 0.99***	2.50 ± 1.40***, †
8‐iso‐PGF2α (pg/mL)	323.73 ± 35.17	524.87 ± 49.67**	790.11 ± 62.14***, ††
IL‐6 (pg/mL)	26.18 ± 5.2	29.98 ± 10.06*	36.58 ± 11.53***, †††
Platelet markers			
CD36 (MFI)	204.54 ± 18.91	305.97 ± 16.94***	384.46 ± 17.21***, ††
Integrin α_IIb_β_3_ (PAC‐1, %)	5.80 ± 1.17	16.97 ± 1.66***	24.06 ± 2.03***, †
Soluble P‐selectin (ng/mL)	20.95 ± 5.77	33.00 ± 10.56***	37.40 ± 10.13***, ††
Comorbidities			
IHD, n (%)	—	30(30.93%)***	79(69.91%)***, †††
HT, n (%)	—	44(45.36%)***	99(87.61%)***, †††
DM, n (%)	—	5(5.16%)	48(42.48%)***, †††
HF, n (%)	—	13(13.40%)***	60(53.10%)***, †††
History of stroke, n (%)	—	—	41(36.28%)***, †††
Treatment			
Warfarin, n (%)	—	30(30.93%)***	41(36.28%)***
ACEI or ARB, n (%)	—	31(31.96%)***	84(74.34%)***, †††
Diuretics, n (%)	—	13(13.40%)*	46(40.71%)***, †††
CCB, n (%)	—	18(18.56%)**	32(28.32%)***
Beta‐blocker, n (%)	—	71(73.20%)***	80(70.80%)***
Statin, n (%)	—	16(16.50%)*	38(33.63%)***, ††

Note

Values are expressed as mean ± SD or number (%). Analyses done by chi‐squared test (for categorical data) or one‐way ANOVA (for continuous data) and post hoc least significant differences t test where appropriate.

Abbreviations: ACEI, angiotensin‐converting enzyme inhibitor; ARB, angiotensin receptor blocker; CCB, calcium channel blocker; DBP, diastolic blood pressure; DM, diabetes mellitus; HF, heart failure; HT, hypertension; IHD, ischaemic heart disease; SBP, systolic blood pressure.

Intergroup differences with least significant differences t test

* *P* < .05.

** *P* < .01.

*** *P* < .001 versus ‘healthy control’.

^†^ *P* < .05.

^††^ *P* < .01.

^†††^ *P* < .001 versus ‘NVAF Patients with low to moderate risk’.

The application of warfarin, calcium channel blockers, beta‐receptor blockers and statins had no significant differences in the high‐risk NVAF patients compared with those with low to moderate risk (*P* > .05); However, the patients in the high‐risk group received more angiotensin‐converting enzyme inhibitor (ACEI) or angiotensin receptor blocker (ARB) compared with those with low to moderate risk (*P* < .01); the healthy controls were free of any medication (Table 1).

There were no significant differences in sex, body mass index (BMI), diastolic blood pressure (DBP), cholesterol, triglyceride (TG), low‐density lipoprotein cholesterol (LDL‐c), high‐density lipoprotein cholesterol (HDL‐c) and platelet counts among healthy controls, NVAF patients at ‘low to moderate risk’ and NVAF patients at ‘high risk’ for stroke (*P* > .05) (Table 1).

### 3.2 Patients in the high‐risk group had significantly higher levels of oxidative stress, inflammation, PMVs and platelet activation

**Figure 1:**
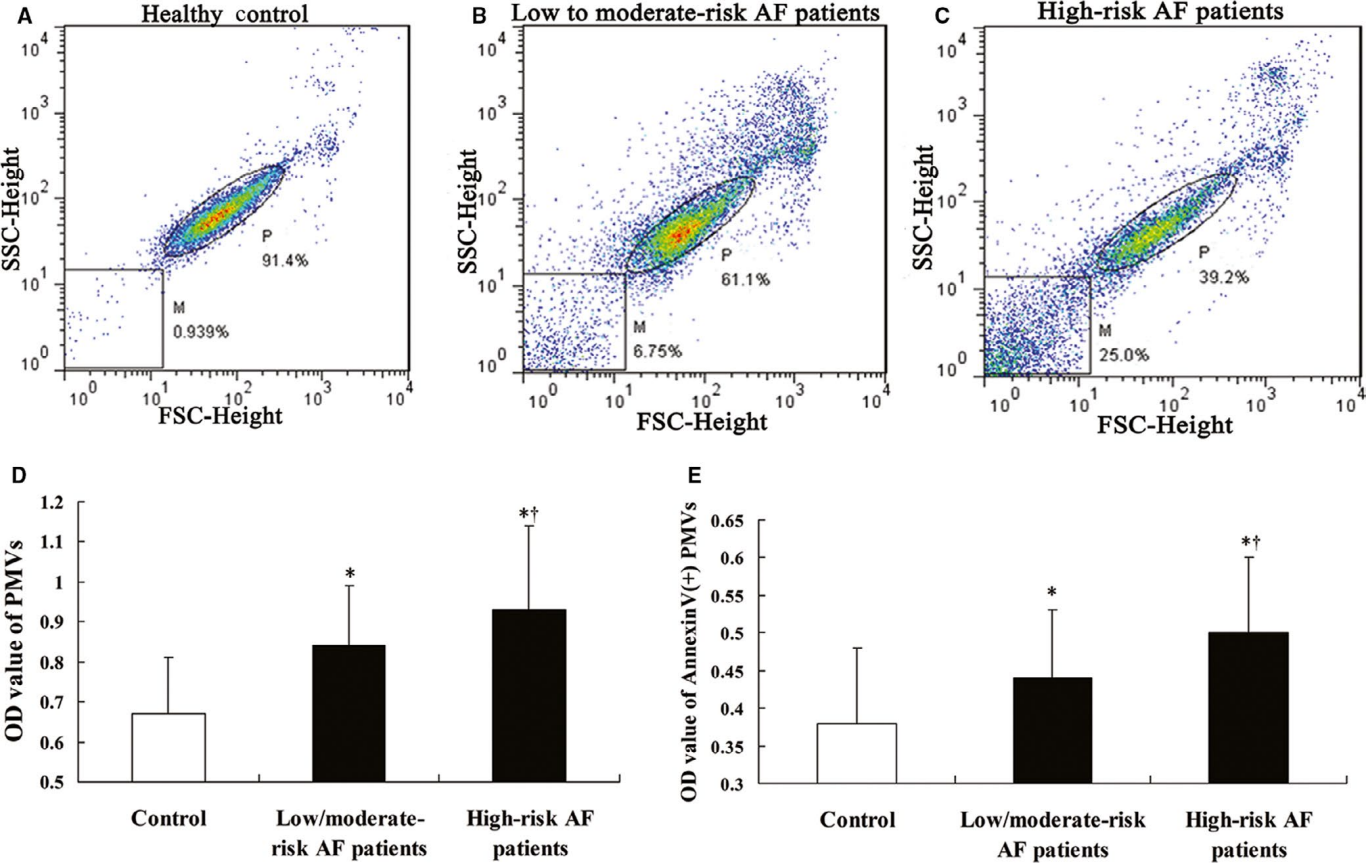
Detection of circulating microvesicles (MVs) in non‐valvular atrial fibrillation (NVAF). A‐C, Forward‐ by side‐scatter profiles of circulating MVs and platelets by flow cytometry. P and M indicate the gates for platelets and MVs, respectively. D‐E, ELISA detection of plasma levels of platelet‐derived MVs (PMVs) (D) and Annexin V‐positive PMVs (E). Data are mean ± SD (n = 33 for control, n = 85 for NVAF at low to moderate risk and n = 100 for NVAF at high risk). *P < .05 compared with control. ^†^ *P* < .05 compared with NVAF at low to moderate risk

Patients at high risk showed significantly higher levels of oxLDL, 8‐isoPGF2α and IL‐6 (*P* < .05) (Table 1), which suggested that these patients had higher levels of oxidative stress and inflammation. Furthermore, patients at high risk showed higher level of PMVs and Annexin V‐positive PMVs compared with those at low to moderate risk (*P* < .05) (Figure 1). Platelet activation in patients was assessed by surface detection of integrin α_IIb_β_3_ and degranulation of P‐selectin, both of which were enhanced in the high‐risk group compared with those at low to moderate risk (*P* < .05). As well, CD36 mean fluorescent intensity (MFI) in platelets was significantly increased in high‐risk patients (*P* < .01) (Table 1).

### 3.3 Platelet function studies with MVs isolated from NVAF patients

#### 3.3.1 Effect of MVs isolated from NVAF patients on platelet activation

**Figure 2:**
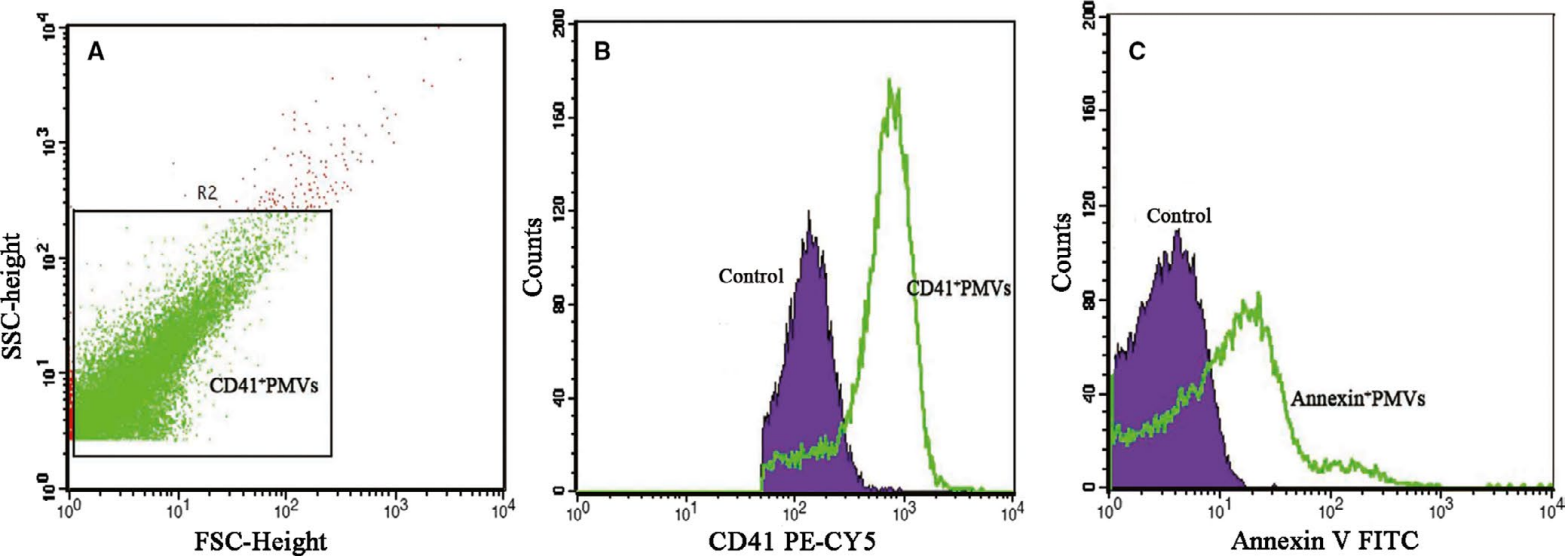
Identification of MVs in patients with non‐valvular atrial fibrillation (NVAF) at high risk of stroke. Flow cytometry of MVs identified by their ability to bind PEcy5‐conjugated anti‐CD41a antibody and FITC‐conjugated Annexin V. A, Forward‐ by side‐scatter profiles of CD41a‐positive events. B, Representative histogram of CD41a‐positive MVs. C: Histogram of Annexin V‐positive MVs

**Figure 3:**
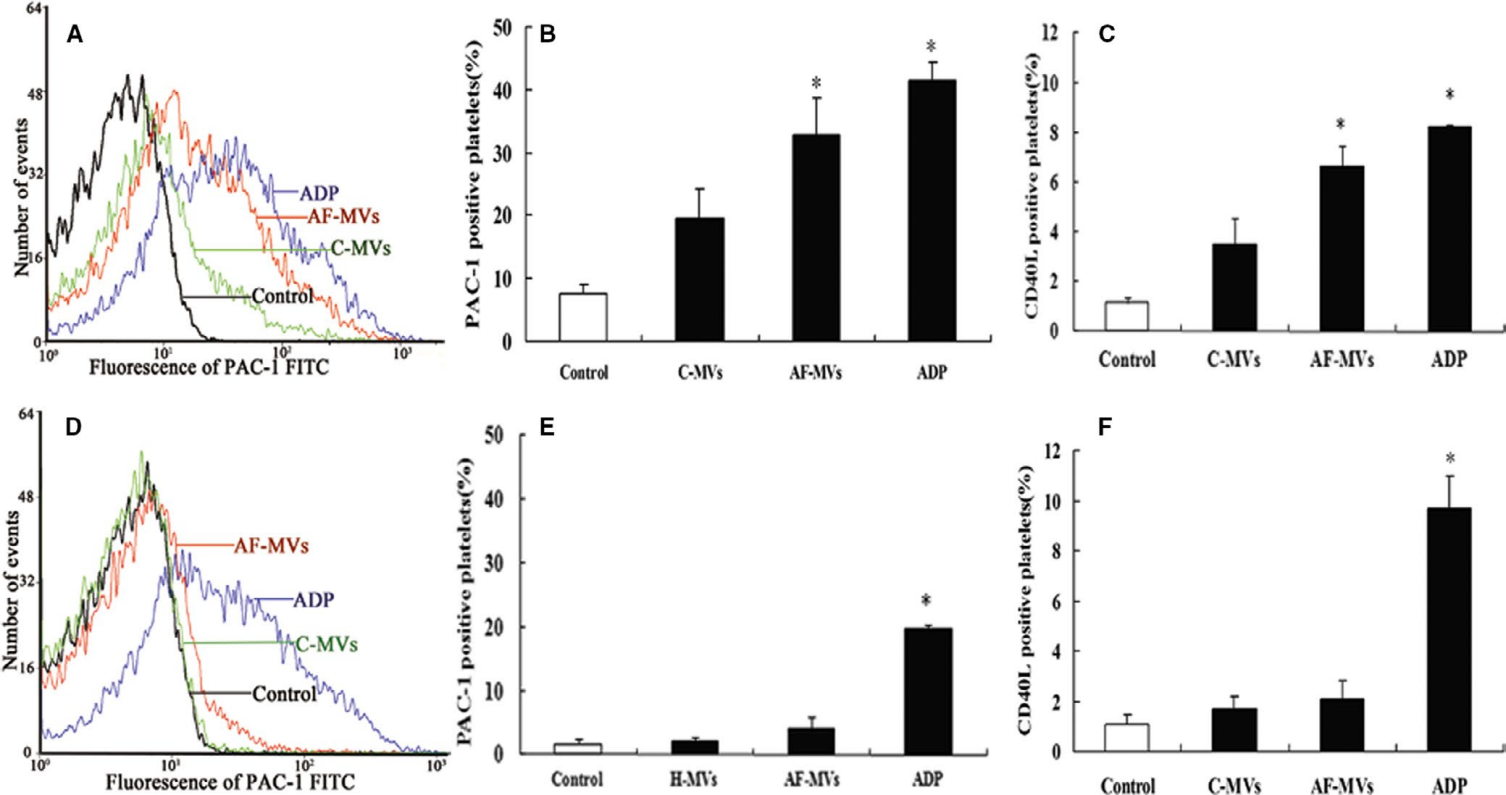
AF‐MVs can activate CD36‐positive but not CD36‐deficient platelets. A‐C, MFI and percentage of PAC‐1, as well as CD40L expression, after incubation of CD36‐positive platelets with AF‐MVs (MVs from patients with non‐valvular atrial fibrillation at high risk of stroke) and C‐MVs (control MVs, from patients at low to moderate risk of stroke) (30 µg/mL) for 30 min. D‐F, Expression of PAC‐1 or CD40L after incubation of CD36‐deficient platelets with AF‐PMVs or C‐MVs (both 30 µg/mL) for 30 min. Data are mean ± SE from at least 3 separate experiments. *P < .05 compared with control

We isolated MVs from the plasma of untreated NVAF patients at high risk of thromboembolism (AF‐MVs) and from NVAF patients at low to moderate risk as control (C‐MVs). The majority of MVs from high‐risk patients were CD41a‐ and Annexin V‐positive (Figure 2). We found enhanced expression of integrin α_IIb_β_3_ and CD40L in normal platelets exposed to AF‐MVs (*P* < .05). Indexes of platelet activation were increased but not significantly in platelets exposed to C‐MVs (Figure 3A‐C).

#### 3.3.2 Activation of platelets by AF‐MVs depends on CD36 and PS

We screened 2 CD36‐deficient donors in the healthy volunteers by flow cytometry. The CD36‐deficient platelets were unable to bind PE‐conjugated anti‐CD36 antibody (Table 2).

**Table 2:** Detection of CD36‐deficient platelets (data expressed as mean fluorescence intensities)

	CD36‐positive platelets (n = 15)	CD36‐deficient platelets (n = 2)
CD36‐PE	487 ± 127***	43 ± 0.1
Isotype control	7 ± 1	8 ± 0.8

Note

Values are expressed as mean ± SD.

*** *P* < .001 versus ‘CD36‐deficient platelets’.

Expression of integrin α_IIb_β_3_ and CD40L in response to AF‐MVs or C‐MVs was not enhanced in CD36‐deficient platelets, whereas the effect of ADP was similar to that in CD36‐positive platelets (Figure 3D‐F).

**Figure 4:**
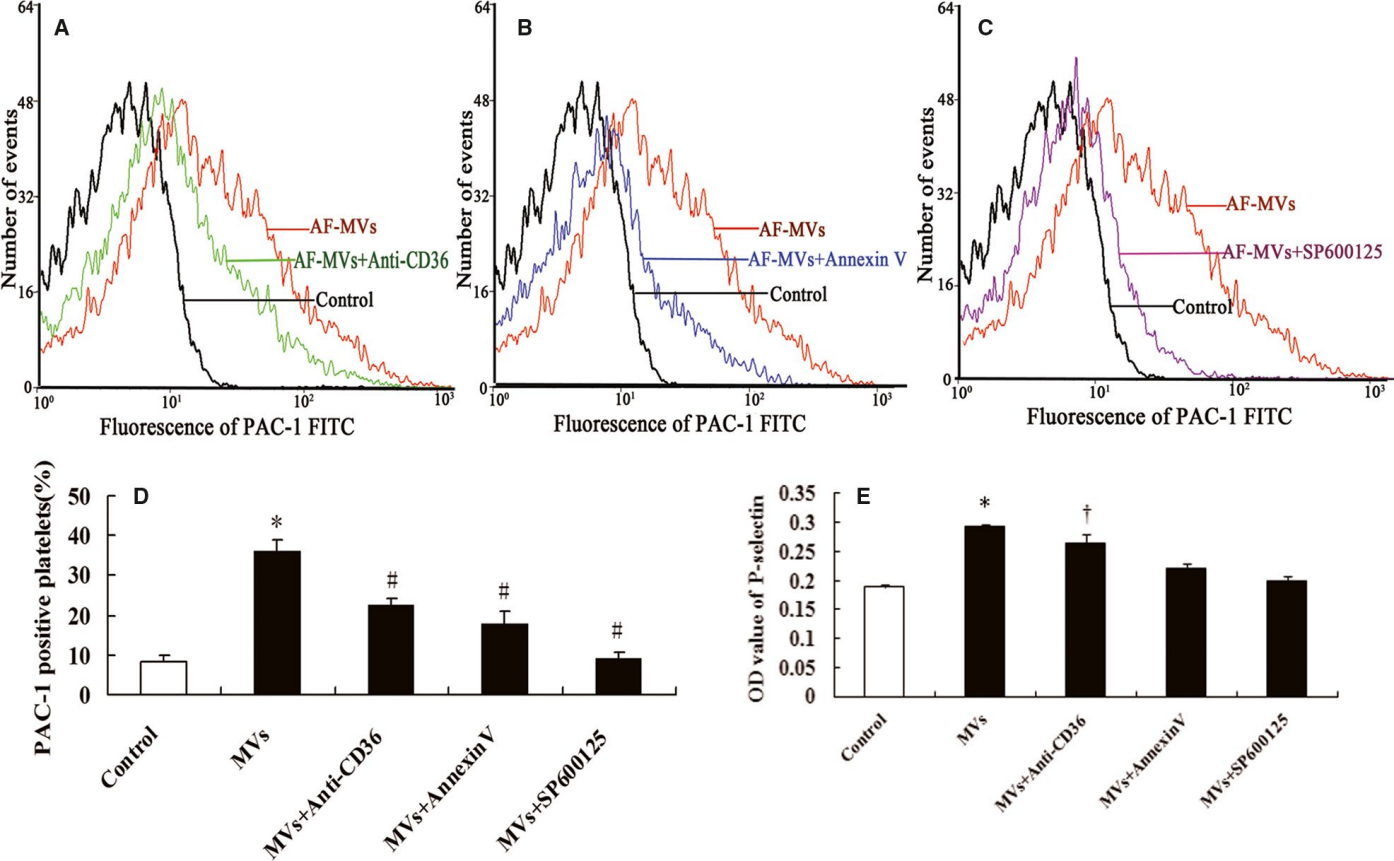
AF‐MV activation of platelets depends on CD36, PS and JNK. Flow cytometry of CD36‐positive platelets treated with 2 µg/mL anti‐CD36 antibody (A, D), 20 µg/mL Annexin V (B, D) or 20 µmol/L JNK inhibitor SP600125 (C, D) before incubation with AF‐PMVs (30 µg/mL). Histograms of A‐C represent MFI, and graph D shows percentage of PAC‐1‐positive platelets. Graph E shows ELISA results of degranulation of platelet P‐selectin. Data are mean ± SE from at least 3 separate experiments. *P < .05 compared with control; ^†^ *P* < .05 compared with MV treatment

Pretreatment with anti‐CD36 antibody or Annexin V decreased platelet activation induced by AF‐MV, as assessed by PAC‐1 binding (*P* < .05) (Figure 4A, B and D) and secretion of P‐selectin (*P* < .05) (Figure 4E).

#### 3.3.3 AF‐MVs upregulate phosphorylation of platelet JNK2 and its upstream activator MKK4

Inhibition of JNK signalling with SP600125 blocked the effect of AF‐MVs on platelet activation, including the expression of integrin α_IIb_β_3_ (*P* < .05) (Figure 4C, D) and secretion of P‐selectin (*P* < .05) (Figure 4E).

**Figure 5:**
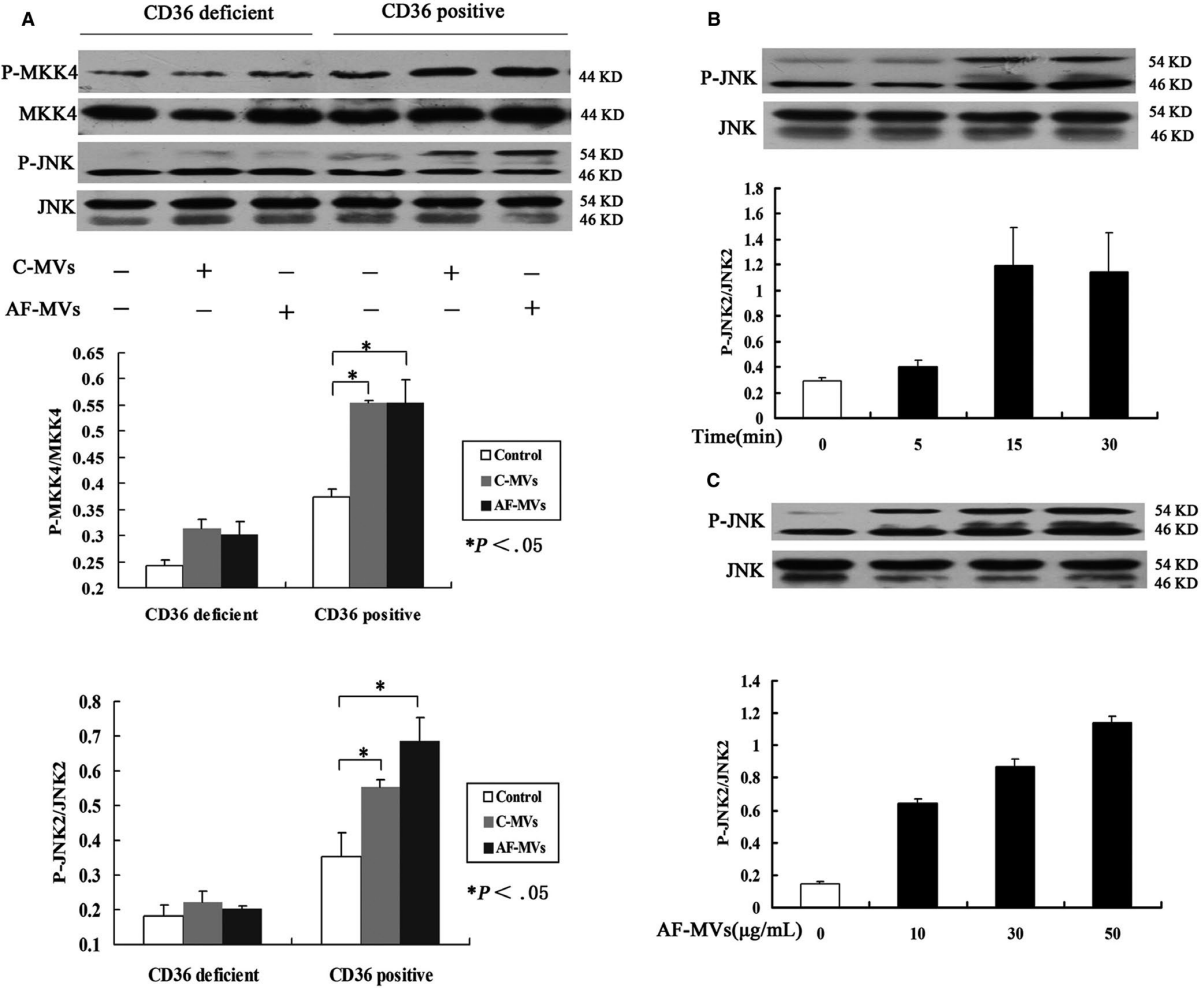
AF‐MVs increase phosphorylation of JNK2 and MKK4 in CD36‐positive platelets. Western blot analysis of phosphorylation of MKK4 and JNK2 in CD36‐positive and CD36‐deficient platelets treated with AF‐MVs (30 µg/mL) by time and dose (A‐C). * *P* < .05 compared with control

To further confirm the role of the JNK pathway in platelet activation, we compared the phosphorylation of JNK2 in CD36‐positive and CD36‐deficient platelets. AF‐MV‐exposed CD36‐positive platelets showed about twofold activation of MKK4/JNK2 as compared with controls (*P* < .05). C‐MV treatment also activated JNK2 but to a lesser extent than AF‐MVs (Figure 5A). CD36‐deficient platelets showed no significant change in MKK4/JNK2 pathway on exposure to AF‐MVs or C‐MVs. The effect of AF‐MVs was time and dose dependent (Figure 5B‐C).

## 4 DISCUSSION

We demonstrated that MVs promoted platelet activation in the setting of NVAF. The engagement of MVs with platelet CD36 plays a critical role in facilitating platelet activation by phosphorylating MKK4/JNK2. Furthermore, we found that the CD36 might be one potential target for preventing prothrombotic state in NVAF.

Platelets provide a catalytic surface for coagulation and bind coagulation factors. Adhesion of the aggregated platelets is the pivotal step of the thrombosis. The molecular mechanisms responsible for platelet activation in NVAF are still obscure, and it is still controversial whether increased platelet reactivity in NVAF is due to the rhythm of atrial arrhythmia or the coexisting proatherogenic factors. The results of AFFIRM and RACE trials did not demonstrate any superiority of a rhythm control versus a rate control strategy on occurrence of ischaemic stroke.^21^ So, the thrombosis is independent of the rhythm of atrial fibrillation. The underlying proatherogenic factors, such as oxidative stress and inflammation, are speculated to be the principal culprit of platelet activation in NVAF.

We speculated that MVs carrying various platelet activation messages may initiate a cascade of platelet activation and might be useful for exploring the underlying mechanisms of the platelet activation in NVAF. A CHADS2 score of more than 2 is used for prediction of thromboembolism. We found that the levels of PMVs enhanced significantly in patients at a high risk for thromboembolism stratified according to the CHADS2 score. The increase of total and Annexin V‐positive PMV levels, as well as the platelet activation, paralleled the CHADS2 score. We found the MVs derived from platelets stimulated by oxLDL ^16^ could promote phosphorylation of the MKK4/JNK2 signal axis and activate platelets by engaging with platelet CD36. To determine the MV roles in platelet activation under AF furtherly, we isolated MVs from plasma of NVAF patients and found the same results. Consistent with our results, Podrez et al showed that platelet‐specific CD36 engagement by oxLDL activated platelets, which contributed to dyslipidemia‐associated thrombus formation; genetic deletion of CD36 protected mice against a hyperlipidemia‐associated prothrombotic phenotype.^22^ Recent studies by Wang et al demonstrated that PS‐exposing microparticles induce procoagulant activity in NVAF.^23^ Similarly, we used Annexin V to mask surface PS exposed on AF‐MVs and found the activation function of AF‐MVs to depend on PS to some extent. Our work and that of others indicated that engagement of PS‐bearing MVs with platelet CD36 triggers platelet activation signals. Then, we explored whether the signal pathway mediated by MV‐CD36 could be a potential target for preventing the prothrombotic state of NVAF. Inhibition of the MKK/JNK pathway may induce unexpected complications because the activation of the MKK/JNK signal pathway regulates a range of biological processes.^24^ In real world of both developed and developing countries, the adherence of NVAF patients to standardized anticoagulant therapy has been a great challenge.^25^, ^26^ Our data make several contributions to the increased knowledge on prothrombotic state in NVAF patients. MVs behave as biological cargo enabling cell interactions, and they may also be a determinant of stroke risk. Human platelet CD36 deficiency is reported in Chinese populations.^27^, ^28^ Nevertheless, these populations do not exist in an obvious haemorrhagic diathesis; blockade of platelet CD36 might be a novel therapeutic strategy for antithrombotic therapy with low risk of bleeding in the setting of NVAF. Furthermore, our results showed that CD36‐deficiency protected platelets from being activated by MVs, implying that CD36 may be the possible candidate target for inhibiting platelet activation.

Our study has limitations. As we obtained peripheral blood other than left atrial appendage, we cannot draw the conclusions regarding the situation within the chambers of the left atrium. Furthermore, the detailed proteomics characteristics of MVs from healthy population, AF patients with low or high risk of stroke, were not clarified in this study, which should be studied in future.

## 5 CONCLUSIONS

Our findings suggest that engagement of the MVs triggers platelet activation in a CD36‐ and PS‐dependent manner in the setting of NVAF, and MKK4/JNK2 signalling pathway might be involved in this procedure. The platelet CD36 may be a potential target for preventing the prothrombotic state of NVAF.

## CONFLICT OF INTEREST

The authors declare that the research was conducted in the absence of any commercial or financial relationships that could be construed as a potential conflict of interest.

## AUTHORS’ CONTRIBUTIONS

Hua Wang and Nian‐Peng Song designed the study, performed the experiments and drafted the article. Jian‐Ping Li and Zhi‐Hao Wang analysed the data and revised the article. Yun Ti and Yi‐Hui Li collected the clinical data and performed the experiments. Wei Zhang and Ming Zhong designed the study and revised the article. All the co‐authors finally contributed to the final approval of the version to be published. All authors read and approved the final manuscript.

## Data Availability

The data used to support the findings of this study are available from the corresponding author upon request.
